# Fluorometric determination of ornidazole by using BSA coated copper nanoclusters as a novel turn off sensor

**DOI:** 10.55730/1300-0527.3321

**Published:** 2021-12-06

**Authors:** Mehmetcan BİLKAY, Hayriye E da ŞATANA KARA

**Affiliations:** Department of Analytical Chemistry, Faculty of Pharmacy, Gazi University, Ankara, Turkey

**Keywords:** Copper nanocluster, ornidazole, fluorescence sensor, pharmaceuticals

## Abstract

A fluorescent probe based on bovine serum albumin stabilized copper nanoclusters (BSA-CuNCs) was developed for the selective and sensitive determination of ornidazole (ORN). The nanoclusters were synthesized via a one-pot hydrothermal process in basic media. The synthesized and characterized BSA-CuNCs have less than 3 nm particle size and exhibited blue emission at 405 nm when excited at 325 nm. Synthesized and characterized nanoclusters were successfully applied as a turn-off fluorescent probe for the determination of ORN in pharmaceutical dosage forms. The quenching mechanism created an was inner filter effect (IFE). The method was linear in the concentration range of 0.52–13.56 **μg** mL^−1^ with a low limit of detection (LOD) 0.01 **μg** mL^−1^. High recovery values (98.5%–102.42%) with low RSD% values (0.25%–2.73%) were obtained. The synthesized nanoclusters can be used as a turn-off probe for ORN determination with their selective, simple, rapid, and inexpensive properties.

## 1. Introduction

Ornidazole, ORN, (1-(3-chloro-2-hydroxy)propyl-2-methyl-5-nitroimidazole or 1-chloro-3-(-2-methyl-5-nitroimidazole-1-yl)propan-2-ol) is a member of third-generation nitroimidazole [1–3] ORN, which has antiprotozoal and antibacterial properties, shows selective activity against anaerobic, microaerophilic bacteria, and protozoa such as Trichomonas and Entamoeba species [2,4]. Therefore, it is used in the treatment of infections such as trichomoniasis, amebiasis, and giardiasis, Helicobacter pylori duodenal ulcers, metronidazole-resistant strains of Trichomonas vaginalis and for prophylaxis in dental and gastric surgery operations [3–5]. Compared to previous nitroimidazoles, ORN has a longer half-life and correspondingly less frequency of dosing. However, the side effects of ORN are mainly nausea, abdominal pain, vomiting, spermatotoxicity, genotoxicity, genomic instability, central nervous system toxicity, and ornidazole-induced autoimmune hepatitis [2]. Analysis of ORN from human plasma, pharmaceutical dosage forms, and other samples have been done with different techniques; these were capillary electrophoresis [4], high performance liquid chromatography (HPLC) used different detectors such as UV [5] and mass spectrometry [6], luminescence spectroscopy [7], and electrochemistry [8]. Although mentioned methods are commonly used and sensitive, they have disadvantages such as low repeatability, need time-consuming sample preparation steps, high operating cost, and need for expert staff. For this reason, a new sensitive, selective, repeatable, simple applying, time-saving, and cheap method for the determination of ORN is needed.

Metal nanoclusters and quantum dots, which are luminescent materials, are very promising compared to organic molecules. However, the use of quantum dots in biological studies can be toxicologically dangerous due to the heavy metals they contain [9, 10]. Metal nanoclusters consisting of several to hundred atoms, they display properties similar to molecules such as spectrochemical and redox properties, magnetism, the HOMO-LUMO transition [11–18]. The most well-known metal nanoclusters (MNCs) are gold (AuNCs), silver (AgNCs), and copper (CuNCs), which are attractive with their size less than 2 nm; on the other hand, the overall size of NCs can be larger with the adding of protective groups to the cluster core [17–20]. Metal nanoclusters have unique features such as low toxicity, quantum-size effect, high photoluminescence efficiency, high stokes shift, water-solubility, photostability, and easy synthesis procedures. Thus, fluorescent metal nanoclusters are used in a broad applications area in various fields such as sensing, biosensing, ion sensing, bio-labeling, single-molecule imaging, chemical catalysis, and pharmaceutical analysis [11–14]. In these studies, because of the interaction of analyte and nanoclusters, the photoluminescence properties of nanoclusters such as luminescence spectrum and/or lifetime change. ---In these studies, the photoluminescence properties of nanoclusters such as luminescence spectrum and/or lifetime change due to the interaction of analyte and nanoclusters. Gold, silver, and copper nanoclusters were generally used for their biocompatible features in these studies. Among them, the synthesis of copper nanoclusters is more economical and easier compared to gold and silver nanoclusters [15]. Different synthesis methods are developed to form nanoclusters based on chemical or photoreduction, chemical etching, ultrasound, and microwave [16]. Some of these methods are using harmful chemicals and needed complicated instruments. Surface ligands not only stabilize NCs but also have a significant effect on their fluorescence properties. Ligands act as a stabilizer that controls the size of NCs, while at the same time, altering the surface function of NCs and, accordingly, their fluorescence properties. DNA, proteins, amino acids, peptides can be used as a template for copper nanoclusters [17]. Recent reports showed that fluorescent copper nanoclusters can be created by proteins from the corresponding metal precursor salt as a precursor. In these studies, bovine serum albumin (BSA), a well-known blood protein, was used forming and stabilizing agent to compose sub-nanosize copper clusters based on reducing Cu precursors.

In addition, BSA has an important place because it has a strong affinity for inorganic metal ions and small molecules, as it contains disulfide bonds and one free cysteine [1]. Besides, the use of BSA as a template also significantly affects the fluorescence properties of CuNCs, which have blue or red emission depending on the synthesis method.

In here, the blue-emitting fluorescent BSA capped copper nanoclusters (BSA-CuNCs) were synthesized via a one-pot hydrothermal process without using any organic, strong reducing agents, and additional stabilizers. Synthesized CuNCs were characterized with different methods such as transmission electron microscopy (TEM), infrared spectroscopy (FT-IR), UV-visible (UV-vis), fluorescence, and X-ray photoelectron spectroscopy (XPS), dynamic light scattering analyses (DLS), and zeta potential measurement. In addition, pre-synthesized NCs were used for the determination of ORN in pharmaceutical dosage forms as a turn-off fluorescent probe. Although there was one study based on using only BSA to determine the ORN [21], in previous studies, the determination of ORN based on interaction with CuNCs in different pharmaceutical forms has not been reported, so the proposed method has a novelty in this field. The developed method has a low detection limit, which indicates the sensitivity of the method and also is repeatable, fast, simple, inexpensive, and eco-friendly.

## 2. Experimental

### 2.1. Chemicals and materials

Ornidazole (ORN), sodium hydroxide, and bovine serum albumin (BSA) were obtained from Sigma–Aldrich Chemical Co. (USA) and used as received without purification. Copper nitrate, phosphoric acid, potassium sulphate, sodium chloride, magnesium chloride, sodium carbonate, potassium nitrate were analytical reagent grades and purchased from Merck (Darmstadt, Germany). The pharmaceutical samples were provided from local pharmacies in Ankara, Turkey. All samples were kept and analyzed at room temperature. Stock ORN solution (1.0 ´ 10^−3^ mol L^−1^) was prepared in deionized water and kept in the refrigerator until analyzing.

Phosphate buffer solutions with different pH values (pH = 2–11; 0.1 M) and sodium hydroxide solution (0.4 M) were prepared using analytical grade reagents and in deionized water. Sodium hydroxide (5 M) was used to adjust the pH value of the buffer solutions. Water used throughout was deionized water (>18 MW .cm).

### 2.2. Instrumentation

In order to obtain TEM images, FEI Tecnai G2 Spirit Biotwin CTEM was used. For FT-IR studies Perkin Elmer Spectrum 400 FTIR / FTNIR spectrometer equipped with a Universal ATR Sampling Accessory (Perkin Elmer Inc., Waltham, Ma, USA) was used and, spectra were reported in cm^−1^. X-ray photoelectron spectroscopic (XPS) analyses were made by using PHI 5000 VersaProbe III multi technique XPS (ULVAC-PHI, Japan). Zeta potential and dynamic light scattering (DLS) analysis were done on a Zetasizer Nano ZS Series, Malvern instrument. UV-Vis spectra were obtained with Specord 50 Plus (Analytik Jena, Germany). Fluorescence measurements were carried out by using the Agilent Cary Eclipse spectrofluorimeter. The excitation wavelength was set at 325 nm and slits were 10.0 nm. Quartz cells with 10 × 10 mm path length were used for all spectroscopic measurements. pH measurements were done with a Mettler-Toledo GmbH (Greifensee-Switzerland) pH meter. All experiments were carried out at room temperature.

### 2.3. Synthesis of BSA stabilized CuNCs

Synthesis of BSA stabilized CuNCs were made by minor modification according to the method of Goswami et al [22]. Briefly, 1 mL, 20 mM aqueous Cu(NO_3_)_2_.3H_2_O was taken into the beaker. Then 5 mL, 15 mg/mL BSA solution was added to this solution and stirred for 3 min at room temperature. After the mixing step, the pH value was adjusted to 12 by adding NaOH. Upon reaching a pH value of 12, the color of the solution changed from pale green to purple. Finally, the mixture was stirred vigorously at 55° C for 7 h. After that time the color of the solution changed to light brown, synthesized nanoclusters were stored in the refrigerator.

### 2.4. Analysis of pharmaceutical dosage forms

In this study, ORN was determined in different pharmaceutical dosage forms such as tablets and injection forms.

To prepare tablet samples, ten tablets containing ORN were weighed and powdered finely in a mortar. An appropriate amount of powder was weighed accurately, taken into the volumetric flask, and diluted with deionized water. Then, it was sonicated for 30 min and centrifuged for 10 min. The obtained supernatant was used for the measurements.

In order to prepare the injection from the sample for injection, 20 μL of the sample was taken and completed with 10 mL of deionized water in the flask, then vortexed for 1 min.

### 2.5. Interaction between ORN and BSA stabilized copper nanoclusters

Before interaction studies, the effect of pH on ORN and BSA-CuNCs was examined. For this purpose, phosphate buffer solutions at different pH values (pH = 2–12; 0.1 M), and 0.1 M NaOH solution (pH = 13) values were tested.

In order to evaluate of interaction between ORN and BSA-CuNCs, a 2.0 mL 0.1 M pH 12 phosphate buffer solution containing an appropriate concentration of BSA-CuNCs was added into the quartz fluorescence cell, and then it was titrated by successive additions of a stock solution of ORN. Titration was done by using a micropipette manually. The fluorescence emission spectra were recorded in the wavelength range of 335–500 nm with an exciting wavelength at 325 nm in the presence and absence of ORN.

The fluorescence intensity changing of nanoclusters as a result of interaction was determined using the ratio of fluorescence intensities of CuNCs in the absence (F_0_) and the presence of ORN (F). All measurements were done with three replicates, and for the calculations obtained, average values were used.

### 2.5. Selectivity studies

In order to evaluate possible interference coming from excipients in pharmaceutical dosage forms and the selectivity of the proposed method, selectivity studies were done. For this purpose, the effect of some possible commonly interfering ions such as potassium, sodium, magnesium, chloride, sulphate, carbonate, nitrate, and excipients such as propylene glycol and ethanol in injection form, starch, and magnesium stearate in tablets on the fluorescence emission intensity of BSA-CuNCs was examined. In this study, the concentrations of ORN and interferents were 36 μM and 360 μM, respectively. The fluorescence emission values of nanoclusters were recorded by adding both ORN and interfering ions/molecules separately on BSA-CuNCs.

## 3. Results and discussion

### 3.1. Characterization of BSA stabilized copper nanoclusters

**Figure 1:**
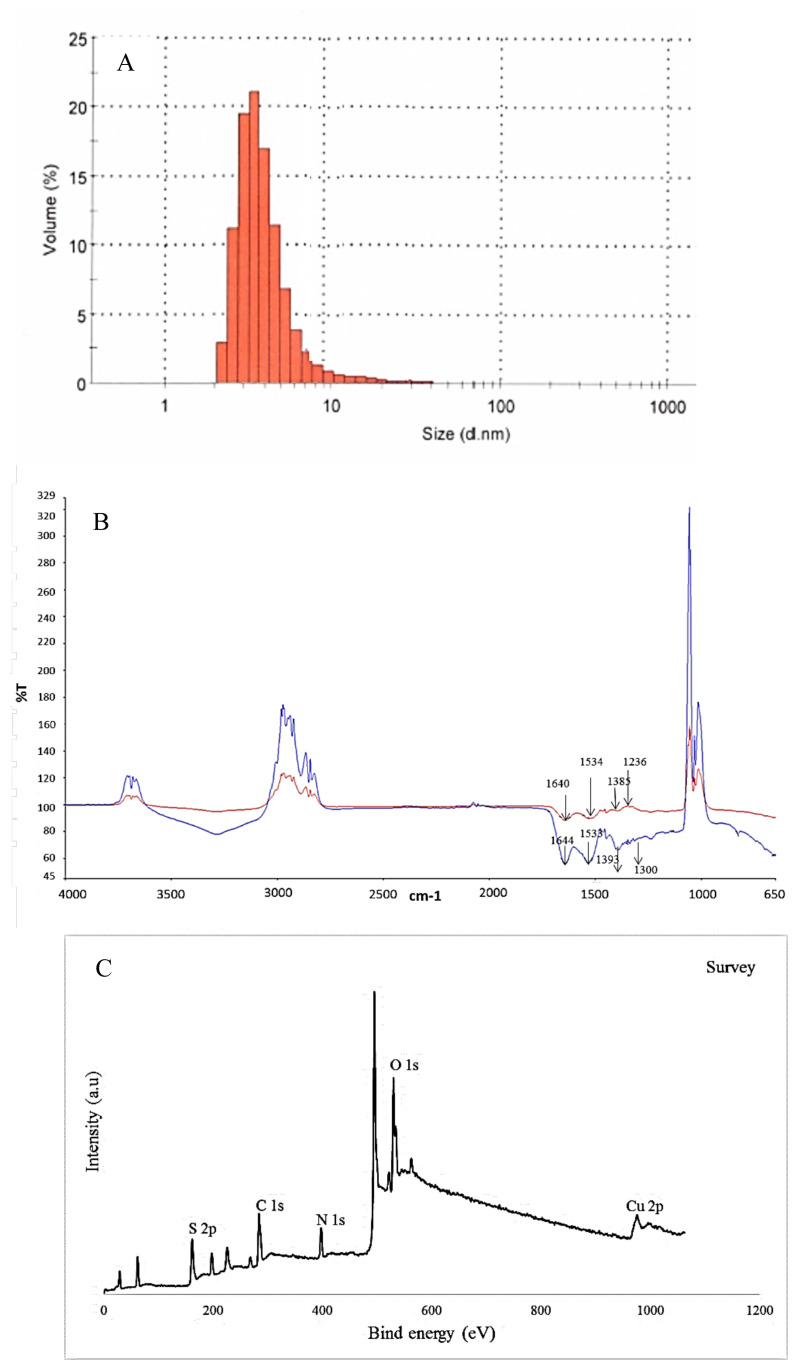
(A) Dynamic light scattering result of BSA-CuNCs, (B) FT-IR spectrum of BSA (red line) and BSA-CuNCs (blue line), (C) The survey XPS spectrum of BSA-CuNCs.

BSA stabilized CuNCs were prepared based on the reduction of copper salts by BSA in the alkaline aqueous solution via the one-pot hydrothermal method. The amino acid residues, which are cysteine, histidine, and tyrosine in BSA, play important and different roles in creating the nanoclusters. The first two of these amino acids can coordinate with metal ions, while the last one can reduce metal ions to form metal NCs. The physical/chemical structure and optical features of prepared BSA-CuNCs were evaluated by using TEM analysis, zeta potential and dynamic light scattering measurements, and different spectroscopic techniques such as UV-vis, fluorescence spectroscopy, and XPS. The TEM image showed that the BSA-CuNCs were in a well-dispersed spherical shape with an average diameter of 2.1 nm particle size, and their distribution was uniform (not shown). Additionally, 3.6 nm particle size, the slightly larger size due to the hydrodynamic radius, was obtained from dynamic light scattering analysis, which was in agreement with TEM analysis (Figure 1A). Moreover, the results of zeta potential analysis showed that synthesized BSA-CuNCs have a negative and high zeta potential value of −31.5 mV in the working buffer solution (pH value at around 12). This negative value represents synthesized nanoclusters have negatively charged and protected the copper nanoclusters from aggregation. The stability studies showed that synthesized nanoclusters were stable for at least 2 months without considerable precipitation in the refrigerator at 4°C.

**Figure 2:**
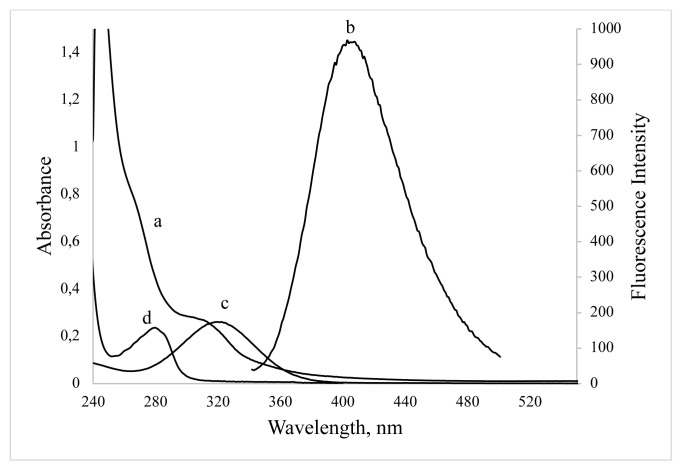
UV–vis absorption (a) and fluorescence emission (b) spectra of BSA-CuNCs, absorption spectra of ORN (c) and BSA (d).

In order to identify the changing of surface groups, the FT-IR spectra of BSA and BSA-CuNCs were recorded (Figure 1B). As shown from the figure, in BSA spectra, the three peaks corresponding to -NH bending (1530 cm^−1^), -CN stretching vibrations (1230 cm^−1^), and amine scissoring (1640 cm^−1^) were observed. Moreover, the bending vibrations peak attributed to the -OH group in carboxylic acids was observed at 1385 cm^−1^. Similar peaks at 1598 cm^−1^ and 1300 cm^−1^ related to -NH bending and -CN stretching were observed in the BSA-CuNCs spectrum indicating interaction with CuNCs and BSA. Besides, -NH scissoring peak was disappeared, which can be explained by the reaction of -NH group with copper during the formation of CuNCs. In addition to these results, although pure BSA has a peak at 280 nm, a weaker peak at 325 nm appears after synthesis of BSA-CuNCs, which can prove the formation of new material in the synthesized environment (Figure 2).

The XPS survey was introduced to explore the components of BSA stabilized CuNCs, and five major peaks at 162, 284, 398, 530, and 932 eV related to S 2p, C 1s, O 1s, N 1s, and Cu 2p were observed (Figure 1C).

In order to evaluate the spectroscopic character of the synthesized BSA stabilized CuNCs, Uv-vis and fluorescence spectra were recorded. Absorption spectrum showed that (Figure 2) no plasmonic absorption peak was observed at the visible wavelengths. This result approves the absence of larger copper nanoclusters in synthesis media. To investigate the fluorescent properties of the synthesized BSA stabilized CuNCs, excitation and emission spectra were recorded, and they showed a maximum emission peak at 405 nm when excited at 325 nm.

### 3.2. ptimization of CuNCs synthesis conditions

**Figure 3:**
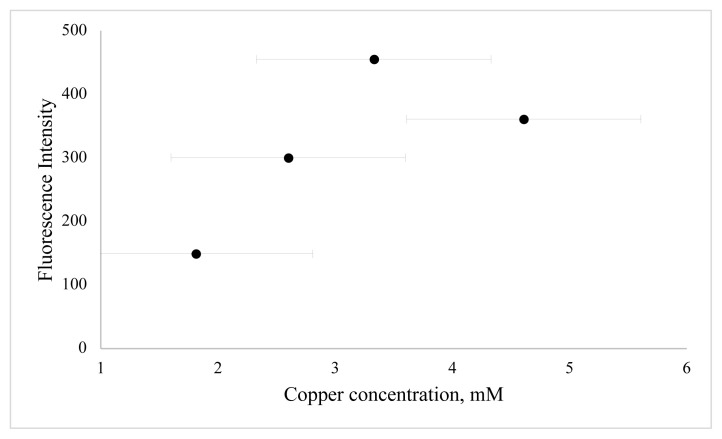
The effect of copper ion concentration on fluorescence emission of BSA-CuNCs. The final metal ion concentrations were 1.81 mM, 2.60 mM, 3.33 mM, and 4.61 mM at keeping BSA concentration at same BSA concentration, i.e. 15 mg/mL.

**Figure 4:**
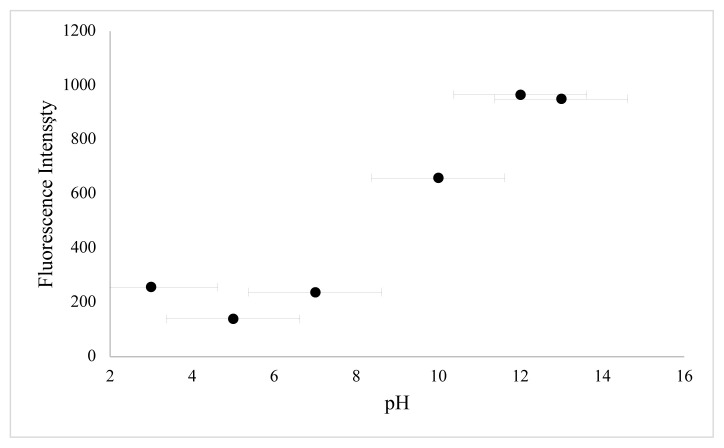
Photoluminescence spectra for BSA-CuNCs synthesized at different pHs values (3–13)

**Figure 5:**
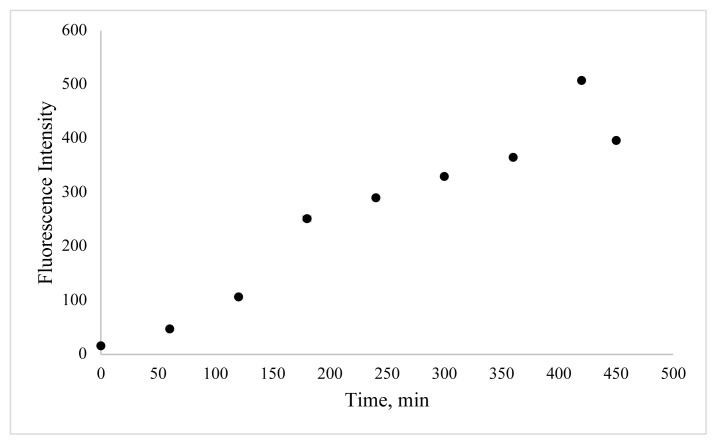
Effect of mixing time on fluorescence emission of BSA-CuNCs.

In this work, BSA was used as a stabilizer for the preparation of CuNCs. Cu^2+^ ions and BSA formed a complex through electrostatic binding and coordination interactions owing to functional groups of BSA such as –NH_2_, -SH. The effect of reaction time, the concentration of metal ion, and pH value were examined to define the optimum synthesis conditions. In order to determine the effect of copper ion concentration, different volumes of copper ion solution were examined. As shown in Figure 3, the emission of clusters is dependent on various metal ion concentrations, and emission reduction occurs as nanoparticles are formed at higher metal ion concentrations. The second key parameter to obtain a high concentration of BSA-CuNCs was pH value. For this purpose, phosphate buffer solutions with different pH values and NaOH solution were examined (Figure 4). Obtained results show that higher fluorescence emission intensity was obtained for the reaction with 3.33 mM Cu(NO_3_)_2_ solution at pH = 12, and this pH was selected. Another crucial step was the mixing time of nanoclusters, which affected the fluorescence properties of BSA-CuNCs. Therefore, the effect of mixing time on the fluorescence emission intensity of BSA-CuNCs from 0 to 7.5 h was examined. As seen from Figure 5, the maximum intensity was obtained at 7th h. The emission signal of BSA-CuNCs decreased after this time, this may be due to overgrowth or aggregation. The experimental results proved this hypothesis because the longer incubation time precipitation occurred, and the color of BSA-CuNCs changed to dark brown.

### 3.3. The selections of experimental conditions

**Figure 6:**
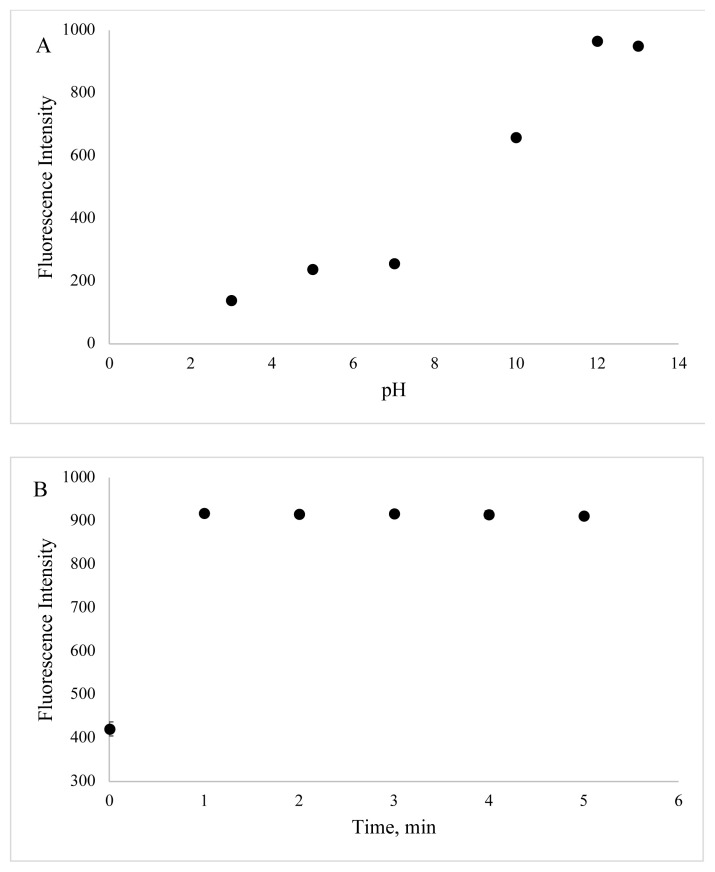
Effect of pH value and incubation time on fluorescence emission of BSA-CuNCs.

In order to identify optimum experimental conditions, the effect of different parameters such as pH value and incubation time on the luminescence of BSA-CuNCs were examined. The obtained results showed that the fluorescence emission of BSA-CuNCs was pH-dependent. As seen from Figure 6A, at the basic area, high emission signal was achieved. This can be attributed to an increase of pair of electrons of BSA at basic pH value, while, at acidic medium, the neutralization of negatively charged BSA-CuNCs was occurred due to high proton concentrations. Therefore, followed experiments were carried out at pH = 12.

The effect of incubation time was also identified from 0 to 5 min, and there was no difference in emission signal after 1 min. Hence, reaction time was chosen as 1 min as the experimental condition (Figure 6B). All optimization figures were given with error bars (Standard deviation, SD, and n=3).

### 3.4. Selectivity of method

**Figure 7:**
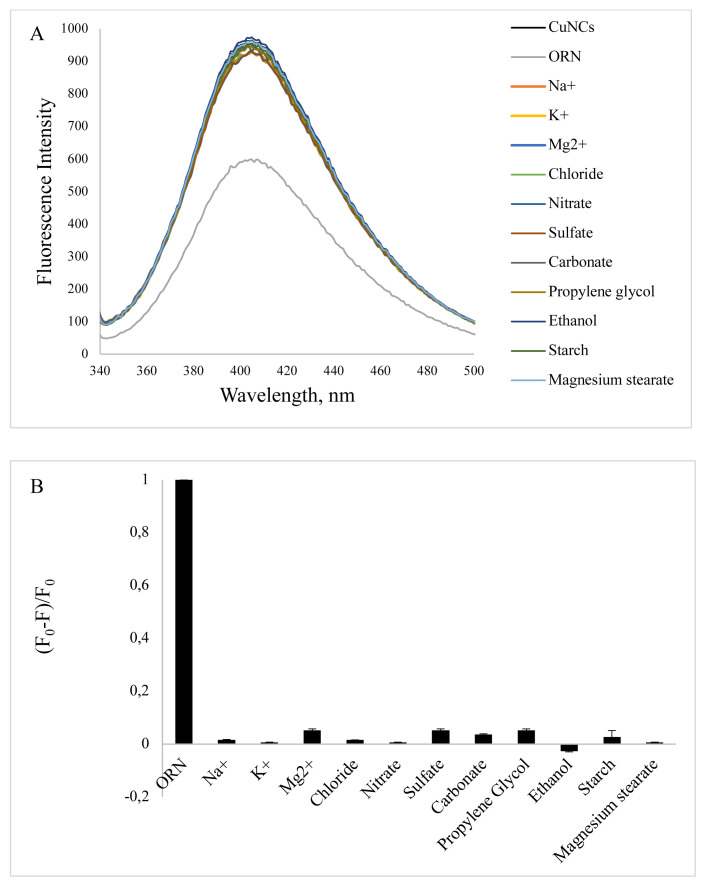
Effect of various interfering substances on the spectra (A) and the normalized (F_0_-F)/ F_0_ of the system (B) The concentration was 36 mM for ORN and 360 mM for interfering substances.

The selectivity of the nanoclusters for the detection of ORN was also evaluated. The effect of excipients such as propylene glycol and ethanol in injection form, starch and magnesium stearate in tablets, and different ions such as K^+^, Na^+^, Mg^2+^, Cl^−^, SO_4_^2−^, CO_3_^2−^, and NO_3_^−^ were tested under optimized experimental conditions. The fluorescence emission of the BSA-CuNCs was measured at 405 nm. The ORN concentration was 36 mM, and excipients / metal ion concentration was 360 mM. As seen from the bar diagram of the fluorescence intensity change (Figure 7), only ORN showed a remarkable quenching of the BSA-CuNCs fluorescence, while the other interfering molecules and ions did not cause a significant response.

### 3.5. Fluorescence quenching mechanism

BSA stabilized CuNCs have excellent chemical and spectroscopic properties such as simple synthesis procedure, suitability to green chemistry, high and stable emission, low toxicity; hence, in this study, they were chosen to be the fluorescent probe for the determination of ORN in pharmaceutical formulations.

**Figure 8:**
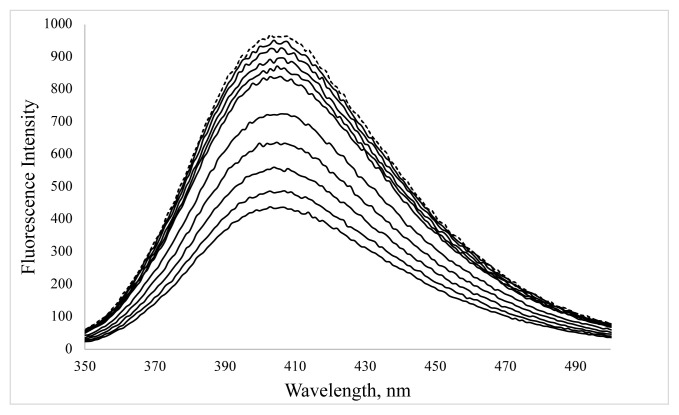
The effect of ORN concentration on fluorescence emission of the BSA-CuNCs. ORN concentration was in the range of 2.35–61.70 μM at pH=12 phosphate buffer (from top to bottom, the dotted line is QDs alone). The inset shows the change of the fluorescence intensity with the increase of the ORN concentration.

The change in the fluorescence spectra of BSA-CuNCs with successive additions of ORN were shown in Figure 8. It can be seen from the Figure the fluorescence intensity of BSA-CuNCs was effectively quenched when the concentration of ORN increases, indicating an interaction between ORN and BSA-CuNCs. The previous study has also shown that the ORN has interacted with tyrosine residues of BSA molecules via hydrophobic and electrostatic forces [21].

Fluorescence quenching mechanisms can occur in several ways: static and dynamic (collisional) quenching, inner filter effect (IFE), and fluorescence resonance energy transfer (FRET). In static and dynamic processes, the fluorophore and the quencher should interact at the ground or excited state. In a static quenching system, both molecules compose a non-fluorescent molecule at the ground state, while in the dynamic (collisional) quenching process, quenchers and fluorophores are interacting in the excited state. Both of them can cause nonradiative relaxation and a decrease in fluorescence emission. The fluorescent resonance energy transfer (FRET) mechanism describes the energy transfer between two light-sensitive molecules, defined as donor and acceptor. For the IFE mechanism to occur, the absorption spectrum of the quencher and the excitation or emission spectrum of the donor must overlap. All these mechanisms can occur simultaneously and competitively.

Figure 2 shows the great overlapping between the absorption spectra of BSA-CuNCs and ORN. UV–Vis absorption spectra of BSA-CuNCs in the presence of ORN were also recorded, and no observable variation was obtained. Moreover, it was noticed that there was a small part of overlap between the emission band of CuNCs and the absorption peak of ORN, which may mean the reabsorption of the NCs emission by ORN. According to Beer–Lambert Law, the molar absorption coefficient of ORN was calculated as 1.9 × 10^4^ at 325 nm wavelength, which demonstrated that it was applicable to sensitive determination by IFE.

In addition, as seen from Figure 8, the fluorescence emission signal of BSA-CuNCs was quenched by adding ORN. Based on the above explanation, this decrease in signal is due to the filtering effect. However, as mentioned earlier, other quenching mechanisms can also happen simultaneously. Stern–Volmer equation defines both static and dynamic quenching mechanisms according to the following formula (Eq. 1) [23].


(1) $${\text{F}}_{0}/\text{F}=1+{\text{K}}_{\text{SV}}\;[\text{Q}]$$

where F_0_ and F indicate the fluorescence intensities of BSA-CuNCs before and after the interaction with ORN, respectively. K_SV_ is the Stern–Volmer quenching constant, and [Q] is the concentration of the ORN. KSV is determined by the slope value of the linearity plot of the F0/F and [Q].

A good linearity was obtained in the range from 2.35 to 61.70 μM with the regression coefficient of r = 0.9910 and equation F_0_ / F = 0.02 [Q] + 0.92 (Figure 4 inset). The obtained results indicated that the static quenching process occurred between the ORN and CuNCs system, and K_SV_ was calculated to be 1.96´10^4^ M^−1^, which shows that interaction between drug and BSA-CuNCs is strong. These results showed that the synthesized nanoclusters can be applied as a fluorescent probe to determine ORN in pharmaceutical samples.

For better identification of the quenching mechanism, Eq. (2) was used to calculate the dynamic fluorescence quenching rate constant, Kq,


(2) $$\text{Kq}={\text{K}}_{\text{D}}/\tau_{0}$$

where τ_0_ is the average lifetime of the fluorophore in the absence of quencher, and the value of τ_0_ is 10^−8^ s for the biopolymer. For collisional quenching, the maximum scattering dynamic quenching constant of various quenchers is 2.0´10^10^ mol L^−1^ s^−1^. On the other hand, the Kq value was calculated as 1.96´10^12^ mol L^−1^ s^−1^, which is greater than the maximum dynamic quenching constant suggesting that there is a nonfluorescent complex formed between the drug and nanoclusters [24]. This result confirms the static quenching mechanism. The site binding model is used to define the number of binding sites (n) and binding constant (K) of ORN with BSA-CuNCs, Eq. (3).


(3) $$\text{log }(({\text{F}}_{0}/\text{F})/\text{F})=\text{log K}+\text{n log }[\text{Q}]$$

These values can be determined by the intercept and slope values of the regression plot of log(F_0_–F)/F vs. log[Q]. The n and K were found as 1.2 and 2.87´10^5^ M^−1^, respectively. The high binding constant indicated a higher affinity between ORN and BSA-CuNCs. The binding constant (K) was also calculated with the Benesi–Hildebrand equation (4).


(4) $${\text{A}}_{0}/(\text{A}-{\text{A}}_{0})={ɛ}_{\text{NCs}}/({ɛ}_{\text{ORN}}-{ɛ}_{\text{NCs}})+{ɛ}_{\text{NCs}}/({ɛ}_{\text{ORN}}-{ɛ}_{\text{NCs}})\times 1/\text{K }[\text{ORN}]$$

where A_0_ and A are the absorbance values of BSA-CuNCs before and after added of ORN, respectively. ɛ_NCs_ and ɛ_ORN_ are the molar absorption coefficients of BSA-CuNCs and their complex with ORN, respectively. In accordance with Eq. (4), the plot of A_0_/(A–A_0_) against 1/[ORN] was constructed using the data from the spectrophotometric titrations, and a linear fitting of the data yielded the binding constant (K), which was 3.04´10^5^ M^−1^ for ORN-CuNCs. The binding constants obtained from fluorescence and UV-vis experiments were compatible.

The degree of repulsion between the similar charges present on the particle surface was described with Zeta potential, and this force keeps the particle from aggregation and, thereby, increases the stability of particles. The measured zeta potential values of prepared particles were −31.5 mV and −15.8 mV in the absence and the presence of ORN, respectively, which might indicate the aggregation of nanoparticles.

### 3.6. Detection of ORN in pharmaceutical forms

The proposed sensor was then applied for the quantitative analysis of ORN in pharmaceutical samples. The good linear correlation obtained using the sensor was achieved in the range of 0.52 to 13.56 μg mL^−1^ with an r value of 0.9934. The linear regression equation was F_0_/F = 0.095 C (μg mL^−1^) + 0.91. The good linearity of the calibration graph and the negligible scatter of experimental points were evaluated by the values of the correlation coefficient and standard deviation values. Several approaches are given in the ICH guidelines to determine the limit of detection (LOD) and limit of quantification (LOQ). Limit of detection (LOD) and limit of quantification (LOQ) values were calculated according to the ICH guidelines by using equations 3 s/m and 10 s/m, where s is the standard deviation of the response, and m is the slope of calibration graph, respectively [25]. LOD and LOQ values were found as 0.01 μg mL^−1^ and 0.04 μg mL^−1^, respectively. In order to evaluate the repeatability and reproducibility of the proposed method, fluorescence intensities of five replicates were measured in the same (intraday precision) and following three days (inter-day precision). The values of inter- and intra-day precision were found as 0.24 and 0.65, respectively. The results summarized in Table 1 indicate a high degree of precision for the proposed method. As indicated by the results (Table 1), the proposed method has a wide dynamic range, good accuracy, and a low detection limit. Hence, it allowed using quantitative determination of ORN in its pharmaceutical forms such as tablets and ampoules. Obtained results have good agreements with labeled values (Table 2). Recovery studies were performed in order to prove the accuracy of the proposed method, by adding samples in an appropriate amount of stock solution of ORN. The recovery values were in the range of 98.50%–102.42% (Table 2). These high recovery values proved the accuracy of the developed method. The proposed method has sensitivity and accuracy for determination from the pharmaceutical dosage forms. Our proposed approach was compared with other separation methods such as capillary electrophoresis [4], HPLC [5], LC-MS [6], fluorescence [7, 21], and electrochemical techniques [8] for the determination of ORN with respect to linear range, LOD, and recovery (Table 3). As seen from the table, the BSA–Cu NCs sensor has a wide linear range, high recovery, low detection limit value, and simplicity when compared with the previous studies. Thus, it holds great practicality for ORN detection in real pharmaceutical samples.

## 4. Conclusion

In this paper, the water-soluble fluorescent BSA-CuNCs were synthesized based on the reaction of copper ions and BSA in basic media by a one-pot hydrothermal process. The obtained results showed that fluorescence of BSA-CuNCs could be quenched due to the IFE mechanism between ORN and NCs. Synthesized and characterized nanoclusters were successfully applied as a fluorescence probe for the determination of ORN in pharmaceutical forms. High recovery values (98.50%–102.42%) with low RSD% values (0.25%–2.73%) were achieved. The proposed method can be used as a promising tool for ORN analysis with its selective, selective, simple, rapid, and inexpensive properties.

## Figures and Tables

**Table 1:** Regression and validation parameters of ORN by using BSA-CuNCs as a fluorescent probe.

Linearity range (μg mL^−^^1^ )	0.52–13.56
Slope	0.095
Intercept	0.91
Correlation coefficient	0.9934
SE of slope	9.17´10^−3^
SE of intercept	0.02
LOD (μg mL^−^^1^)	0.01
LOQ (μg mL^−^^1^)	0.04
Intra-day precision (RSD%)	0.24
Inter-day precision (RSD%)	0.65

SE: Standard error

RSD: Relative standard deviation

LOD: Limit of detection

LOQ: Limit of quantitation

**Table 2:** Dosage form and recovery analysis of ORN.

Tablet (%)		Ampoule (%)	
99.70		101.06	
103.40	Mean 99.04	100.24	Mean 100.63
96.95	RSD % 2.73	100.56	RSD % 0.25
95.51		100.64	
99.63		100.64	
Added (μg mL^−^^1^)	Found (μg mL^−^^1^)		Recovery (%)
1.65	1.69		102.42
3.3	3.25		98.50
4.95	4.90		99.00

**Table 3:** Comparison of the proposed method with different techniques reported for the determination of ORN.

Method	Linear range (μg mL^−^^1^)	LOD (μg mL^−^^1^)	Recovery (%)	Ref.
Capillary electrophoresis	25–250	1.80	96.2–105.2	4
HPLC	0.03–5.08	0.01	99.5–104.1	5
LC-MS	0.03–10.0	0.03	96.5–102	6
Fluorescence-Graphene QD	0.16–6.59	0.05	104.5	7
Electrochemical	0.15–22.1 22.1–54.9	0.05	99.3–100.4	8
Fluorescence	0.40–80	0.11	99.7–100.8	21
Fluorescence-BSA-CuNCs	0.52–13.56	0.01	98.5–102.4	This work
